# Influence of Androgen Deprivation Therapy on the PD-L1 Expression and Immune Activity in Prostate Cancer Tissue

**DOI:** 10.3389/fmolb.2022.878353

**Published:** 2022-06-28

**Authors:** Ulrich Sommer, Celina Ebersbach, Alicia-Marie K. Beier, Gustavo B. Baretton, Christian Thomas, Angelika Borkowetz, Holger H. H. Erb

**Affiliations:** ^1^ Institute of Pathology, Universitätsklinikum Carl Gustav Carus Dresden, Dresden, Germany; ^2^ National Center for Tumor Diseases Partner Site Dresden and German Cancer Center Heidelberg, Dresden, Germany; ^3^ Tumor and Normal Tissue Bank of the University Cancer Center (UCC), University Hospital and Faculty of Medicine, Technische Universität Dresden, Dresden, Germany; ^4^ Department of Urology, Technische Universität Dresden, Dresden, Germany; ^5^ Department of Urology, Mildred Scheel Early Career Center, Medical Faculty and University Hospital Carl Gustav Carus, Technische Universität Dresden, Dresden, Germany

**Keywords:** checkpoint inhibitors, immune therapy, PCA, ADT, tumour microenvironment (TME)

## Abstract

Immune checkpoint inhibitors have become a promising new therapy for cancer treatment. However, due to prostate cancer’s high heterogeneity and immune-suppressive tumour microenvironment, clinical trials with immune checkpoint inhibitors for prostate cancer resulted in low or no response. This descriptive and retrospective study investigates the influence of androgen deprivation therapy (ADT) on PD-L1 expression and CD8^+^ T-cell tumour infiltration and activity in primary prostate cancer tissue. Therefore, immunohistochemistry was used to assess PD-L1, CD8^+^ T-cell, and the immune activation marker Granzyme B (GrB) in PCa tissue before and under ADT. In line with previous studies, few prostate cancer tissues showed PD-L1 expression and CD8^+^ T-cell infiltration. However, PD-L1 expression levels on tumour cells or infiltrating immune cells above 5% generated an immune-suppressive tumour microenvironment harbouring hypofunctional CD8^+^ T-cells. Moreover, analysis of a longitudinal patient cohort before and under ADT revealed that ADT increased hypofunctional CD8^+^ T cells in the tumour area suggesting a tumour immune milieu optimal for targeting with immunotherapy.

## Introduction

Since their FDA and EMA approval, immune checkpoint inhibitors have played an essential role in the therapy of various cancer entities (Robert, 2020). These inhibitors block specific checkpoint proteins from binding with their partner proteins. Especially blocking of the cytotoxic T lymphocyte-associated protein-4 (CTLA-4), the programmed death (PD)-1 T cell receptor, and its ligand PD-L1 have shown to be promising targets to activate the immune system to attack cancer cells. Therefore, inhibition of these proteins by specific antibodies activates immune cells such as the cytotoxic CD8^+^ T cells, subsequently targeting the tumour cells (Raskov et al., 2021). In recent years PD1/PD-L1 inhibitors were established in multiple guidelines alone or in combination with chemotherapies as first or second line of treatment (Yu et al., 2019; Robert, 2020).

Prostate cancer (PCa) is the most common cancer in men, next to lung and colon cancer, and PCa’s development and progression are highly dependent on androgens (Isaacs, 2018; Sung et al., 2021). Most patients are diagnosed with local confined PCa and are treated with radiotherapy or radical prostatectomy (Mottet et al., 2021). For locally advanced or metastatic PCa, androgen deprivation therapy (ADT) by chemical castration or orchiectomyADT combined with antiandrogens or taxan based chemotherapy are the gold standard (Mottet et al., 2021). However, the treatment inevitably leads to the development of castration-resistant PCa (CRPC), which is currently incurable (Santer et al., 2015; Mottet et al., 2021). PCa’s high intra and intertumoral heterogeneity is one of the main reasons for treatment failure and the challenge of defining an efficient treatment strategy for CRPC (Frame et al., 2017; Li et al., 2019; Brady et al. Therefore, new therapeutic strategies are mandatory to manage CPRC.

Due to its relatively low somatic mutation frequency and few tumour-infiltrating T cells, PCa belongs to the class of immunologically cold tumours (Elia et al., 2018). Therefore, PCa is discussed to be resistant to immune checkpoint therapies. This issue is also represented in multiple clinical trials with CTLA-4 and PD-1/PD-L1 inhibitors showing only low overall survival benefits in patients with CRPC (Beer et al., 2017; Antonarakis et al., 2020; Sharma et al., 2020; Sweeney et al., 2020). Furthermore, recent studies identified patients who failed treatment with novel hormonal therapies (NHT) abiraterone acetate and enzalutamide as a sub-group of patients having good clinical activity of PD-1/PD-L1 inhibitors alone or in combination with docetaxel (Agarwal et al., 2020; Antonarakis et al., 2020; Appleman et al., 2021; Fizazi et al., 2021). These observations are evaluated in the currently running Phase III KEYNOTE-921 trial assessing the efficacy and safety of the PD-L1 and PD-L2 inhibitor pembrolizumab combined with docetaxel in metastasised CRPC (mCRPC) patients pretreated with NHT (Petrylak et al., 2021).

Tumour-infiltrating lymphocytes, especially cytotoxic CD8^+^ T-cells, are associated with response to checkpoint inhibitors in several tumour entities such as melanoma and ovarian carcinoma (Almeida et al., 2020; Raskov et al., 2021). However, in PCa, the immune suppressive CD4^+^ FOXP3+ CD25^+^ T cell subpopulation dominates the tumour-infiltrating lymphocytes (Bethmann et al., 2017). In recent studies, ADT has been reported to modulate the composition of the immune milieu in the tumour microenvironment (Almeida et al., 2020). Therefore, ADT before radical prostatectomy promotes CD8^+^ T-cells infiltration (Mercader et al., 2001; Gannon et al., 2009; Sorrentino et al., 2011).

As the immune milieu in the tumour microenvironment plays an essential role in responding to immune checkpoint inhibitors, a deeper understanding of the influence of modulation of the androgen receptor on the immune milieu signalling pathways is mandatory. Therefore, this descriptive and retrospective pilot study aims to analyse the influence of ADT on PD-L1 expression and CD8^+^ T-cell tumour infiltration and activity. To this end, immunohistochemistry was used to assess the expression of PD-L1, CD8^+^, and the immune activation marker Granzyme B (GrB) in PCa tissue before and under ADT.

## Materials and Methods

### Patient Material

The patient’s cohort has previously been described in Sommer et al., 2022 and the samples were selected from the Tumor and Normal Tissue Bank of the University Cancer Center Dresden (Ebersbach et al., 2022; Sommer et al., 2022). The cohort of this study contained 116 tissue specimens of 97 PCa patients undergoing transurethral resection of the prostate (TURP) recruited from 2011 to 2020. Some of these patients received multiple TURPs. Of these patients, five patients were selected for the longitudinal patient cohort. Details information about the cohort is displayed in supplementary table 1. The Ethics Committee of the Medical University Dresden approved the use of archived material (Study no. EK59032007, 06.03.2007). According to statutory provisions, written consent was obtained from all patients and documented in the Carl Gustav Carus Dresden medical hospital database.

### Immunohistochemistry

The tissue blocks were cut in serial sections of 1–2 µm thickness and deparaffinised with a BenchMark XT (Ventana Medical Systems, Oro Valley, United States), followed by heat-induced epitope retrieval. The PD-L1 (Clone E1L3N; LOT: 18) antibody (Cell Signaling, Danvers, Massachusetts, United States) has been used in a 1:100 dilution for staining, followed by counterstaining with hematoxylin, dehydration, and mounting of the slides. The PD-L1 clone E1L3N is one of the most commonly used antibodies for PD-L1 and exhibited high specificity for PD-L1 testing (Xu et al., 2021).

### Data Evaluation of PD-L1

PD-L1-stained slides were scored for PD-L1 immune cell (IC)-positivity (percentage of tumour area covered by stained IC) and PD-L1 tumour cell (TC)-positivity (percentage of positive PD-L1 TC in the tumour area). The data was analysed by senior pathologists specialising in PD-L1 evaluation in various tumour entities. PD-L1 IC-positivity was defined as staining in granulocytes, lymphocytes, macrophages and dendritic cells of any intensity within the tumour area. PD-L1 TC-positivity was defined as membranous PD-L1 staining of any intensity in all detectable tumour cells on the slide. In addition, the combined positive score (CPS) was determined, which is calculated as the total number of PD-L1 positive TC and IC in the tumour area divided by the total number of viable TC multiplied by 100% (Frederick et al., 2020). PD-L1 expression values ≥ 1% and a CPS ≥1 were defined as positive. For the CD8 and granzyme B double staining, the primary CD8 antibody (clone C8/144B (1:10), Dako) and the primary granzyme B antibody (clone GrzB-7 (1:10), Dako) were blended, followed by counterstaining with hematoxylin, dehydration and mounting of the slides.

### Data Evaluation of CD8 and GrB

The immunoreactions for the T-cell population were digitised with a Panoramic Scan II (3DHistech LTD., Budapest, Hungary), followed by a pathologist’s manual quantification of the cells. Quantification was done within the peritumoral stroma. To assess peritumoral stroma, the hot spot method was used and up to three high power fields (HPF) with the highest density of immune cells within the peritumoral stroma were chosen and analysed. Since this immunohistochemical test is a double reaction, CD8^+^ cells were quantified first (red chromogen) and, in a second step, those cells that also showed GrB were counted from the CD8^+^ cells (brown chromogen).

### Statistics

Prism 9.3.1 (GraphPad Software, San Diego, CA, United States) was used for all statistical analyses. Differences between treatment groups were analysed using ordinary one-way ANOVA or Student’s t-test. *p*-values of ≤0.05 were considered statistically significant. Kaplan-Meier estimate has been used for overall survival analysis. The Pearson correlation coefficient (r) has been calculated and interpreted suggested by Schober et al. for correlation analysis (Schober et al., 2018). All differences highlighted by asterisks were statistically significant as encoded in figure legends (*: *p* ≤ 0.05; **: *p* ≤ 0.01; ***p ≤ 0.001).

## Results

### Influence of PD-L1 Expression on CD8^+^ T-Cell Infiltration and Activity in Primary Prostate Cancer Tissue.

**FIGURE 1:**
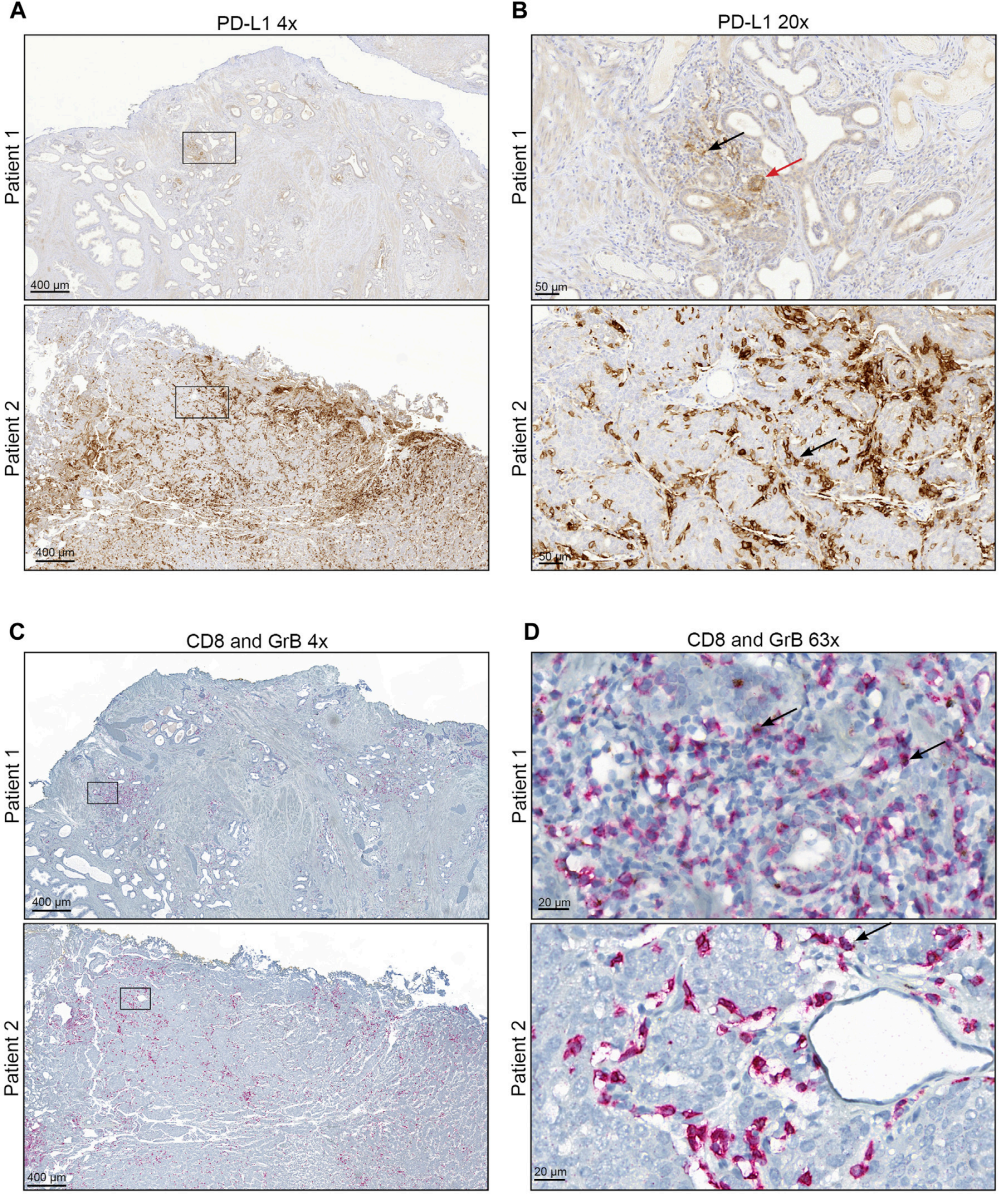
Representative staining of PD-L1, CD8^+^, and GrB (A + B) Immunohistochemical staining for PD-L1 of representative PCa areas obtained with **(A)** ×4 objective (Scale bar = 400 µM) and **(B)** ×20 objective (Scale bar = 50 µM). The red arrow mark represents PD-L1 positive tumour cells. Black arrows mark representative PD-L1 positive infiltrating immune cells. (C + D) Immunohistochemical staining for CD8 and Granzyme B of representative PCa areas was obtained with **(A)** ×4 objective (Scale bar = 400 µM) and **(B)** ×63 objective (Scale bar = 20 µM). CD8 is represented by red staining and Granzyme B by brown staining. Black arrows mark representative CD8 and Granzyme B positive infiltrating immune cells.

**FIGURE 2:**
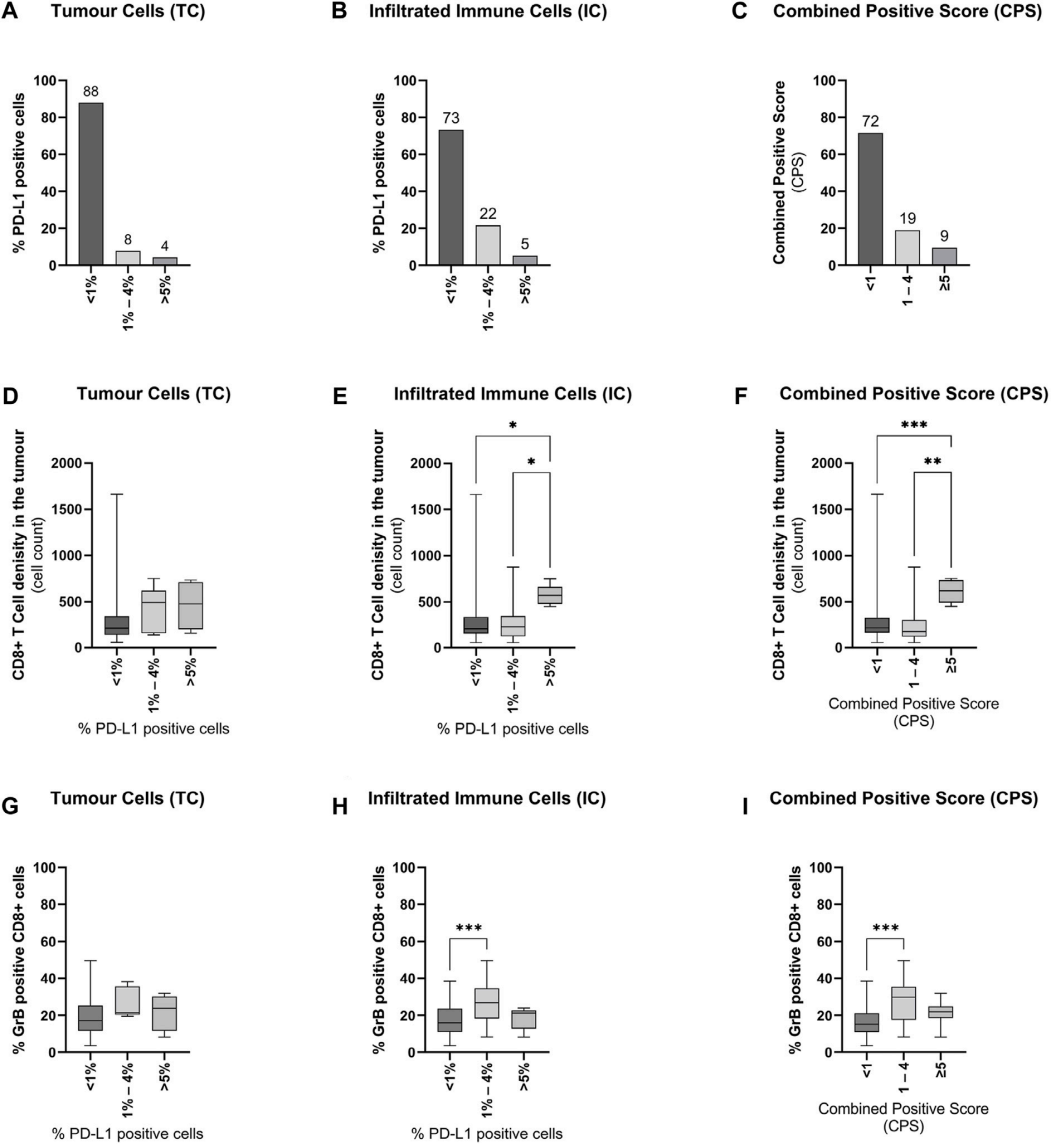
Influence of PD-L1 expression on CD8^+^ T-cell infiltration and activity in primary prostate cancer tissue (A + B + C) Graphical illustration of the distribution of PD-L1 expression on **(A)** tumour cells, **(B)** infiltrating immune cells, and the resulting combined positive score (CPS) in all examined tissue specimens (*n* = 116). Numbers are displayed as column bar blots. (D + E + F) Graphical illustration of the associations of CD8^+^ T-cells infiltration with **(D)** PD-L1 expression on tumour cells, **(E)** PD-L1 expression on tumour-infiltrating immune, and **(F)** the combined positive score in all examined tissue specimens (*n* = 116). Values are expressed as Box Whisker Plot (min to max). (G + H + I) Graphical illustration of the associations of CD8^+^ T-cells activity with **(G)** PD-L1 expression on tumour cells, **(H)** PD-L1 expression on tumour-infiltrating immune cells, and **(I)** the combined positive score in all examined tissue specimens (*n* = 116). Values are expressed as Box Whisker Plot (min to max). All differences highlighted by asterisks were statistically significant (*: *p* ≤ 0.05; **: *p* ≤ 0.01; ***p ≤ 0.001).

Representative immunohistochemical images for PD-L1, CD8^+^, and GrB are displayed in Figure 1. In primary tumours, PD-L1 expression (≥1%) on TC and IC could be evaluated in 12 and 27% of 117 cases, respectively (Supplementary Figure 1A+B). The most positive PD-L1 tumour samples have 1–4% PD-L1 positive cells (Figure 2A+B). Resulting from these values, 28% of the specimens have a positive CPS (≥1, Supplementary Figure 1C) accumulating at a CPS between 1 and 4 (Figure 2C). Furthermore, the PD-L1 positive TC (≥1%) showed an increased number of infiltrating CD8^+^ T-cells (Figure 2D, Supplementary Figure 1D). Samples with a positive CPS (≥1) also revealed an increase in infiltrating CD8 + T cells in the tumour areas (Supplementary Figure 1F). An increased level of PD-L1 expression in TC or IC (≥5%) and CPS (≥5) could be associated with a significant increase in infiltrating CD8^+^ T-cells (Figure 2E+F). To assess the CD8^+^ T-cells activity in the tumour areas, a GrB staining was performed. In general, the presence of positive PD-L1 (≥1%) cells is associated with an increase in CD8^+^ T-cells activity (Supplementary Figure 1G-I). The number of PD-L1 positive TC had only marginally influence on the infiltrated CD8^+^ T-cells activity (Figure 2G). Only a low number of PD-L1 positive infiltrated CD8^+^ T-cells (1–4%) was associated with a significant activity increase (Figure 2H). In line with this observation, a low CPS (1–4) was associated with a significant activity increase of CD8^+^ T-cells (Figure 2) and a CPS higher than ≥5 was associated with only a marginal activity increase. Correlation analysis revealed no positive or negative relationship between CD8^+^ T-cell tumour infiltration, CD8^+^ T-cell activation, or PD-L1 expression (Supplementary Figure S2). However, the PD-L1 expression on TC and IC correlated with the CPS.

### Influence of PD-L1 Expression on CD8^+^ T Cell Infiltration and Activity on Overall Survival.

**FIGURE 3:**
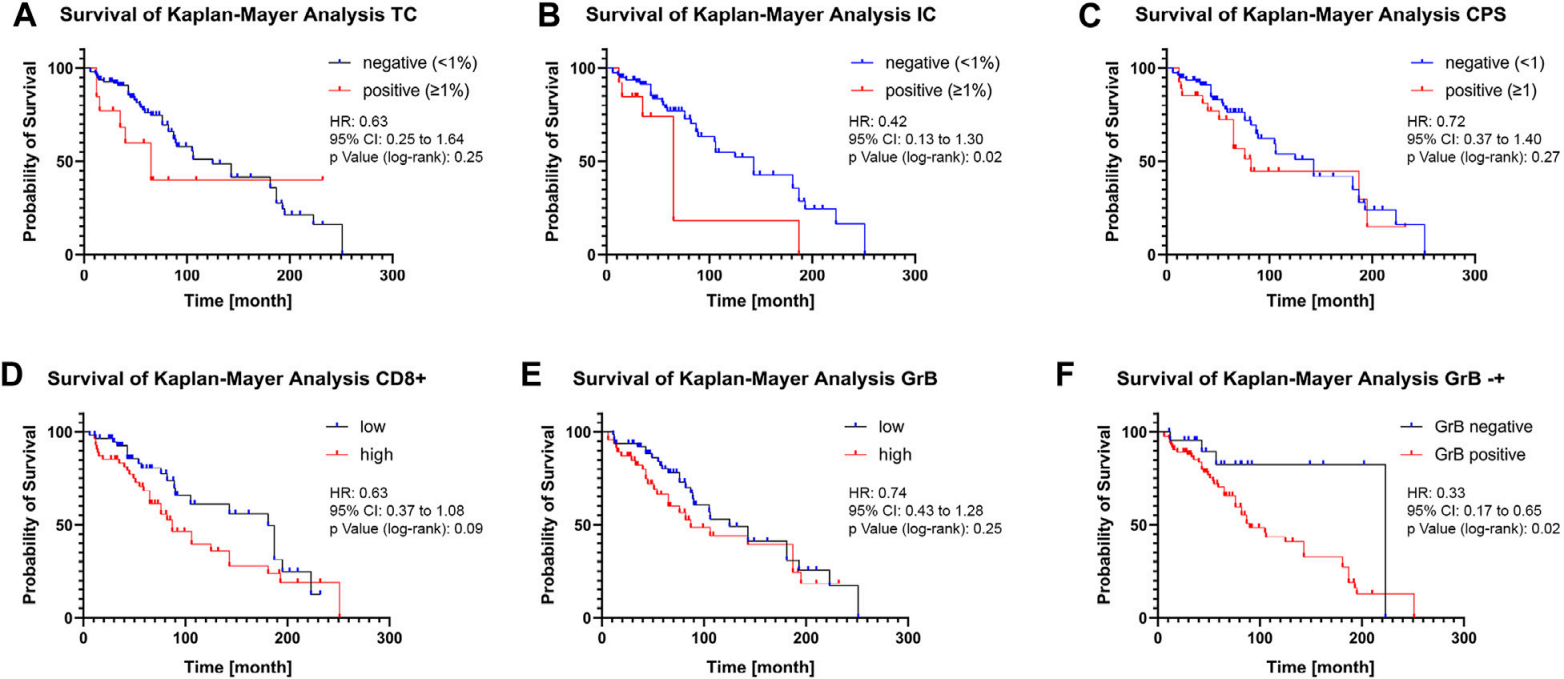
Influence of PD-L1 expression on CD8^+^ T-cell infiltration and activity on overall survival (A + B + C) Kaplan-Meier analysis of overall survival according to positive and negative PD-L1 expression on tumour cells **(A)**, on tumour-infiltrating immune cells **(B)**, or combined positive score **(C)**. 1% PD-L1 positive expression in TC or IC was used as the threshold. (D + E) Kaplan-Meier analysis of overall survival according to high and low CD8^+^ T Cell infiltration **(D)** or CD8^+^ T-cell activity **(E)**. The median infiltration number of CD8^+^ T-cell activity was used as the threshold. **(F)** Kaplan-Meier analysis of overall survival according to absence or presence of GrB staining. Data is displayed together with the hazard ratio (HR), 95% confidence interval (CI), and the *p*-value calculated using the log-rank (Mantel-Cox) test.

Kaplan-Meier survival analysis was performed to assess the influence of PD-L1 expression on CD8^+^ T-cell infiltration and CD8^+^ T-cell activity in tumour areas on overall survival (OS). Kaplan-Meier survival analysis revealed no significant difference in OS for PD-L1 negative (<1%) TC patients and PD-L1 positive (≥1%) TC patients (Figure 3A). For patients with PD-L1 positive TC, a significant decrease in median OS from 143 months to median OS of 65 months could be revealed (Figure 3B). The CPS status had also no significant effect on OS (Figure 3C). Patients with a low CD8^+^ T-cell tumour infiltrate had a median OS of 181 months, whereas patients with a high infiltrate had a median OS of 87 months (Figure 3D). Low or high CD8^+^ T-cell tumour activity had no significant effect on OS (Figure 3E). However, patients with no CD8^+^ T-cell activity in the tumour area had a median OS of 223 months, significantly decreasing to 90 months in patients with CD8^+^ T-cell activity (Figure 3F).

### Influence of ADT on PD-L1 Expression on TC and IC

**FIGURE 4:**
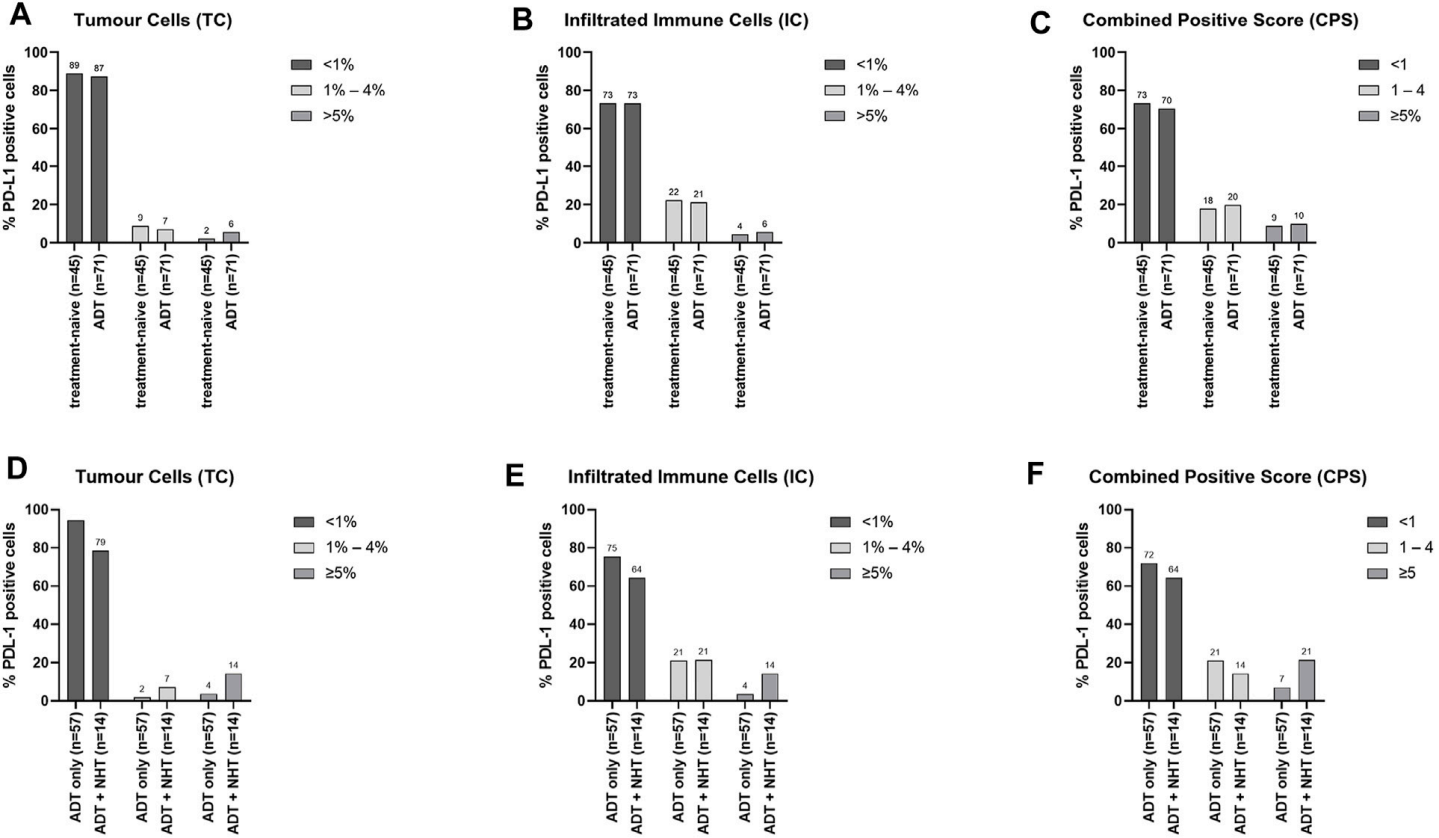
Influence of ADT on PD-L1 expression on TC and IC (A + B + C) Graphical illustration of the influence of ADT on the distribution of PD-L1 expression on **(A)** tumour cells (TC), **(B)** infiltrating immune cells (IC), and the resulting **(C)** combined positive score (CPS). The “treatment-naïve” cohort included 45 samples and the “ADT” cohort 71 samples. (D + E + F) Graphical illustration of the influence of additional treatment with NHT on the distribution of PD-L1 expression on **(D)** tumour cells (TC), **(E)** infiltrating immune cells (IC), and the resulting **(F)** combined positive score (CPS). The “ADT only” cohort included 57 samples and the “ADT + NHT” cohort 14 samples. Numbers are displayed as column bar blots.

To assess the influence of ADT on PD-L1 expression, the cohort was sub-grouped into treatment-naïve and ADT. TC showed higher PD-L1 levels under ADT (Supplementary Figure 3A, Figure 4A), whereas IC PD-L1 expression was unchanged (Supplementary Figure 3B, Figure 4B). The CPS also revealed an increase in the ADT specimens compared to the treatment-naïve specimens (Supplementary Figure 3C, Figure 4C). As patients treated with novel hormonal therapies (NHT) abiraterone acetate and enzalutamide responded well to PD-1/PD-L1 inhibitors, the ADT cohort was further sub-divided into an “ADT only” cohort and an “ADT + NHT” cohort (Agarwal et al., 2020; Antonarakis et al., 2020; Appleman et al., 2021; Fizazi et al., 2021). Therefore, the ADT only cohort represents specimens under buserelin, degarelix, triptorelin, or leuprorelin treatment. The ADT + NHT represents specimens under ADT combined with abiraterone or enzalutamide. Compared to the ADT only cohort, the ADT + NHT cohort revealed an increase in PD-L1 positive cells on TC and IC (Supplementary Figure 3D+E). This increase is reflected explicitly in the increase in IC and TC with over 5% PD-L1 expression (Figure 4D+E). In line with these results, the CPS also increases after ADT (Supplementary Figure 3F, Figure 4F).

### Influence of ADT on the CD8^+^ T-Cell Tumour Infiltrates

**FIGURE 5:**
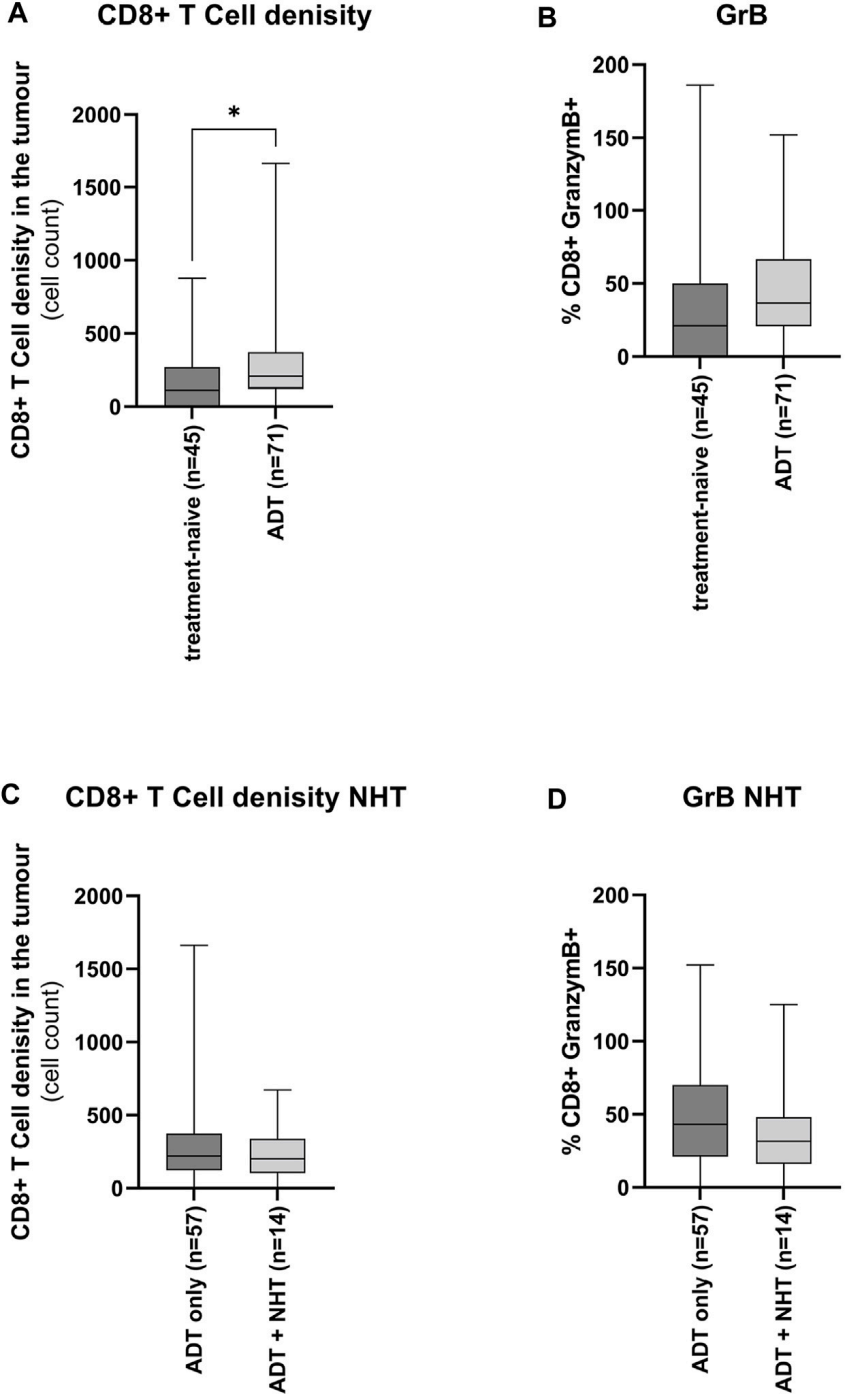
Influence of ADT on the CD8^+^ T-cell tumour infiltrates (A + B) Graphical illustration of the influence of ADT on CD8^+^ T-cells **(A)** infiltration and **(B)** activity. The “treatment-naïve” cohort included 45 samples and the “ADT” cohort 71 samples. (C + D) Graphical illustration of the influence of ADT of additional treatment with NHT on CD8^+^ T-cells **(C)** infiltration and **(D)** activity. The “ADT only” cohort included 57 samples and the “ADT + NHT” cohort 14 samples. Values are expressed as Box Whisker Plot (min to max). All differences highlighted by asterisks were statistically significant (*: *p* ≤ 0.05).

Reduction of androgen levels has been reported to increase CD8^+^ T-cell infiltration in benign prostate hyperplasia (Fan et al., 2014; Yang et al., 2017). Therefore, the influence of ADT on CD8^+^ T-cell tumour infiltration and activity has been assessed. Tumour tissue under ADT harboured a significantly higher number of CD8^+^ T-cells than the treatment-naïve tumour tissue (Figure 5A). However, there was no significant increase in CD8^+^ T-cell activity (Figure 5B). Furthermore, this increase in ADT tissue was treatment independent (Figure 5C+D).

### Influence of ADT on PD-L1 Expression, CD8^+^ T-Cell Infiltration, and CD8^+^ T-Cell Activity in a Longitudinal Patient Cohort.

To validate if the previous results are due to ADT or tumour heterogeneity, PD-L1 expression, CD8^+^ T-cell infiltration, and CD8^+^ T-cell activity were assessed in an exploratory study using a longitudinal patient cohort. ADT altered PD-L1 expression heterogeneously (Supplementary Figures 4A–C). In contrast, 4 out of 5 patients increased CD8^+^ T-cell infiltration under ADT (Supplementary Figure 4D), whereas CD8^+^ T-cell activity was marginally changed (Supplementary Figure 4E).

## Discussion

The immune system has been utilised successfully to fight highly immunogenic cancers such as lung cancer and renal carcinoma (May et al., 2020; Robert, 2020). However, due to its immune-suppressive tumour microenvironment, PCa is categorised as a so-called cold tumour. The immune-suppressive tumour microenvironment of PCa is caused by the infiltration of regulatory T-cells, tumour-associated macrophages, myeloid-derived suppressor cells, and cytokines (Shiao et al., 2016; Stultz and Fong, 2021). Therefore, tumour-infiltrating lymphocytes, especially cytotoxic CD8^+^ T-cells, are less attracted to the tumour side or are directly inactivated. Consequently, results of immunotherapy trials in PCa have been generally disappointing so far. However, a sub-group of patients who failed NHT demonstrated an excellent response to PD-1/PD-L1 inhibitors (Agarwal et al., 2020; Antonarakis et al., 2020; Appleman et al., 2021; Fizazi et al., 2021). Moreover, ADT has been reported to modulate the composition of the immune milieu in the tumour microenvironment by promoting CD8^+^ T-cell infiltration (Mercader et al., 2001; Gannon et al., 2009; Sorrentino et al., 2011). This study aimed to investigate if ADT changes PD-L1 protein distribution as well as the CD8^+^ T-cell infiltration and activity or if alterations are due to the high PCa heterogeneity.

The PCa cohort used in the present study included only 12% positive PD-L1 cases. This low percentage in positive cases is in line with previously published data reporting TC specific PD-L1 expression in 7–11% of cases (Martin et al., 2015; Baas et al., 2017; Calagua et al., 2017; Haffner et al., 2018). In addition, consistently with the data published by Haffner and others, most of these cases had a PD-L1 expression on TCs between 1 and 4% (Haffner et al., 2018). PD-L1 expression on tumour infiltrating lymphocytes was reported in up to 15% of the cases (Ebelt et al., 2009; Baas et al., 2017; Haffner et al., 2018). Here, PD-L1 expression on IC was 27%, and in line with the TCs, most of these cases had a PD-L1 expression on ICs above 1 and 4%. However, correlation analysis revealed no relationship between the PD-L1 expression on TCs and ICs. However, increased levels of PD-L1 on ICs and TCs were associated with a higher number of tumours infiltrating CD8^+^ T-cells. This finding is controversial with previous studies reporting a negative correlation between PD-L1 expression and the number of CD8-positive T-cells (Ye et al., 2009; Gabrielson et al., 2016; Xie et al., 2016). On the other hand, Dang and others reported that the numbers of CD8^+^ T-cells increased in PD-L1-positive tumours compared to PD-L1-negative ones (Deng et al., 2021). The groups hypothesised that the number of CD8^+^ T-cells does not directly represent the number of active T cells, especially in PD-L1-positive tumours, and that CD8-positive T-cells may be in a hypofunctional state. This hypothesis is supported by the findings presented here. Even if an increased number of CD8^+^ T-cells could be found in the high PD-L1 tumour areas, they only showed low levels in GrB, a marker for CD8^+^ T-cell activity.

PD-L1 protein expression on TC and IC is the most frequently studied biomarker for checkpoint inhibitors (Davis and Patel, 2019). The PD-L1 thresholds were variable both within and across tumour types and indications, including approvals at 1, 5, and 50% PD-L1 expression on TC and 1 and 5% on IC. Due to the disappointing response in clinical trials, no positive threshold in PCa has been defined yet. However, most studies used ≥1% to determine PD-L1 positivity (Li et al., 2019). This study showed increased infiltration of hypofunctional CD8^+^ T cells above 5% PD-L1 expression on TC and IC. This result suggests that PD-L1 expression ≥5% forms an immune-suppressive tumour microenvironment and should be considered a possible threshold for PD-L1 positivity.

CPS has been introduced to eliminate the choice between tumour and immune cell PD-L1 expression as a predictive biomarker and especially also to reflect the positive predictive value for response to immune checkpoint inhibitor therapy of both tumour and immune cells. However, the score has been rarely applied in PCa (Palicelli et al., 2021). CD8^+^ T-cell tumour infiltration and activity differences could be more clearly dissected using CPS. Therefore, the CPS should also be considered more carefully for PCa.

Several studies have reported that high PD-L1 expression influences the OS of cancer patients (Aguiar et al., 2017; Steiniche et al., 2017; Svensson et al., 2019). For example, high PD-L1 expression was associated with a prolonged OS in gastric cancer, whereas high PD-L1 expression in oesophagal cancer was associated with a shorter OS (Svensson et al., 2019). In addition, activated cytotoxic T-lymphocytes (GrB positive T-cells) are a strong and independent prognostic marker for OS in patients with diffuse large B-cell lymphoma (Muris et al., 2004). Kaplan-Meier survival analysis performed in this study revealed a significant longer OS in patients positive for PD-L1 or GrB. As most specimens with PD-L1 levels of 1–4% had the highest CD8^+^ T-cells activity, it can be concluded that patients with a tumorigenic tumour have a survival advantage compared to patients with a tumour-suppressive tumour microenvironment and immune cell infiltrate. Disadvantages of a tumour-suppressive tumour microenvironment in survival have previously been reported in ovarian and endometrial cancers (Ni et al., 2021).

ADT modulates PD-L1 expression and CD8^+^ T-cells infiltration in the tumour microenvironment (Mercader et al., 2001; Gannon et al., 2009; Sorrentino et al., 2011; Agarwal et al., 2020; Antonarakis et al., 2020; Appleman et al., 2021; Fizazi et al., 2021). This observation could be validated in the present study showing that ADT increased PD-L1 expression and CD8^+^ T-cells infiltration in the tumour microenvironment. Especially treatment with NHT led to increased PD-L1 expression and CD8^+^ T-cell infiltration. However, due to the simultaneous increase in PD-L1 and CD8^+^ T-cell infiltration, there was no change in CD8^+^ T-cells, as shown by the GrB staining. To validate if the rise in the measured parameters is not caused by PCa heterogeneity, a longitudinal patient cohort was assessed on changes in PD-L1 expression, CD8^+^ T-cell infiltration, and CD8^+^ T-cell activity. This cohort included treatment-naïve PCa specimens and specimens under ADT from the same patient. In this longitudinal cohort, changes in PD-L1 expression by ADT could not be confirmed. However, approximal 80% of the patients have an increase in CD8^+^ T-cell infiltration. This result strengthens the hypothesis that patients receiving immune checkpoint inhibitors would benefit from pretreatment with NHT, such as abiraterone and enzalutamide, as they harbour more hypofunctional CD8^+^ T-cells in the tumour areas.

## Conclusion

This retrospective descriptive study analysed PD-L1 and CD8^+^ T-cell infiltration in primary tumour tissue received by TURP. In line with previous studies, only a low tissue number showed PD-L1 expression and CD8^+^ T-cell infiltration. However, positive PD-L1 expression levels above 5% generated an immune-suppressive tumour microenvironment harbouring hypofunctional CD8^+^ T-cells. Moreover, a longitudinal patient cohort analysis before and under ADT revealed that ADT increased hypofunctional CD8^+^ T-cells in the tumour area, suggesting an inactive tumour immune milieu. Therefore, despite the heterogeneity of the PCa’s tumour microenvironment, it has been shown here that ADT can enforce a more homogeneous immune milieu to prime for immunotherapy. However, the main limitation of this study is the small number in the longitudinal patient cohort. Also, information on the patients’ tumour mutational burden (TMB) would be a nice add on as TMB had been connected to immunotherapy response rates (Yarchoan et al., 2017). Moreover, information about regulatory lymphocytes would be desirable as they have a pivotal role in maintaining the homeostasis of the immune system and self-tolerance. Therefore, these results should be seen as a pilot study which should serve as a basis for future investigations.

## Data Availability

The original contributions presented in the study are included in the article/Supplementary Material, further inquiries can be directed to the corresponding authors.
