# Supplementary figures and images for: Influence of Androgen Deprivation Therapy on the PD-L1 Expression and Immune Activity in Prostate Cancer Tissue
Source: Front Mol Biosci. 2022 Jun 28;9:878353. doi: 10.3389/fmolb.2022.878353 (PMC9273856; doi:10.3389/fmolb.2022.878353)

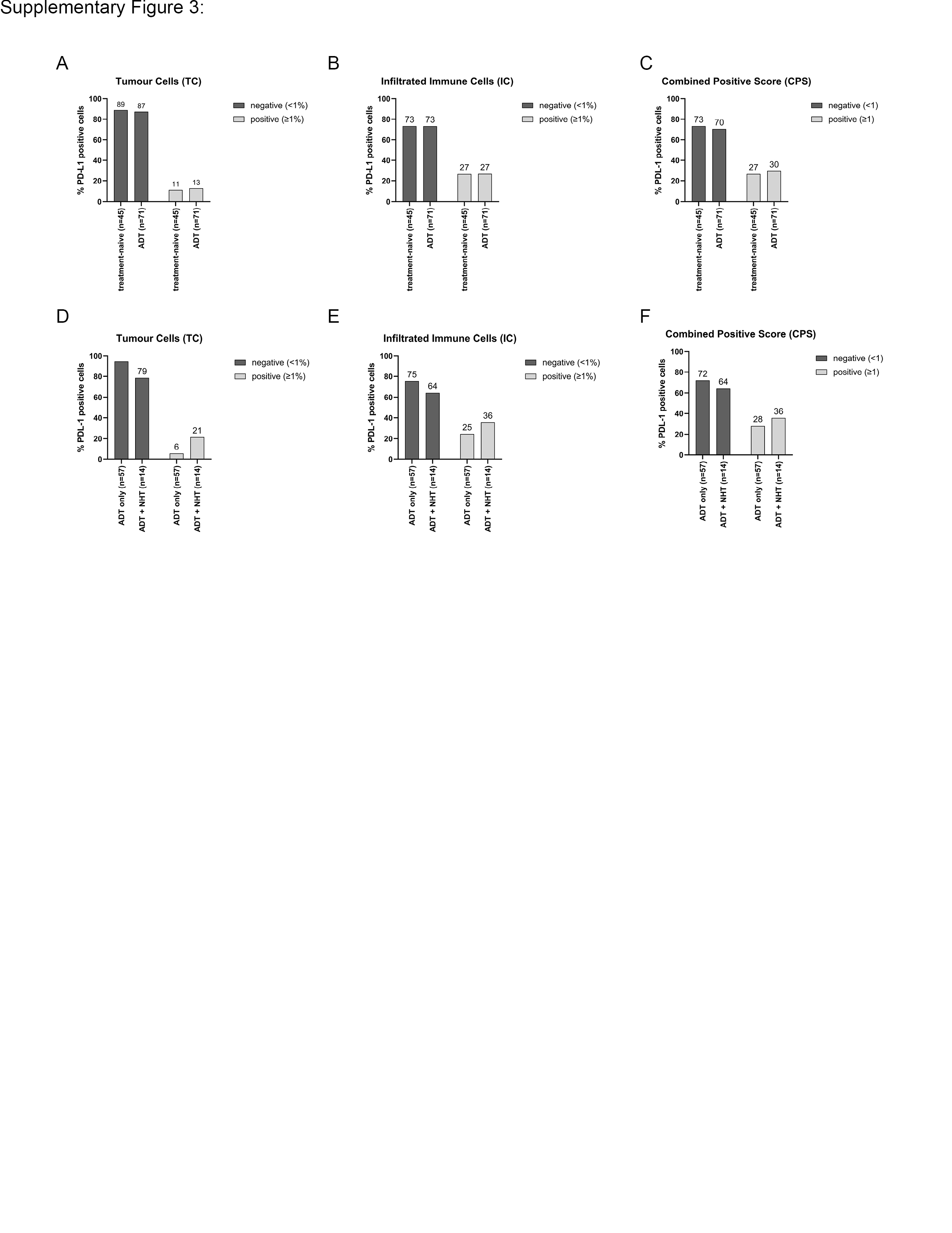

Supplement: Supplementary file 1 [file Image3.JPEG]

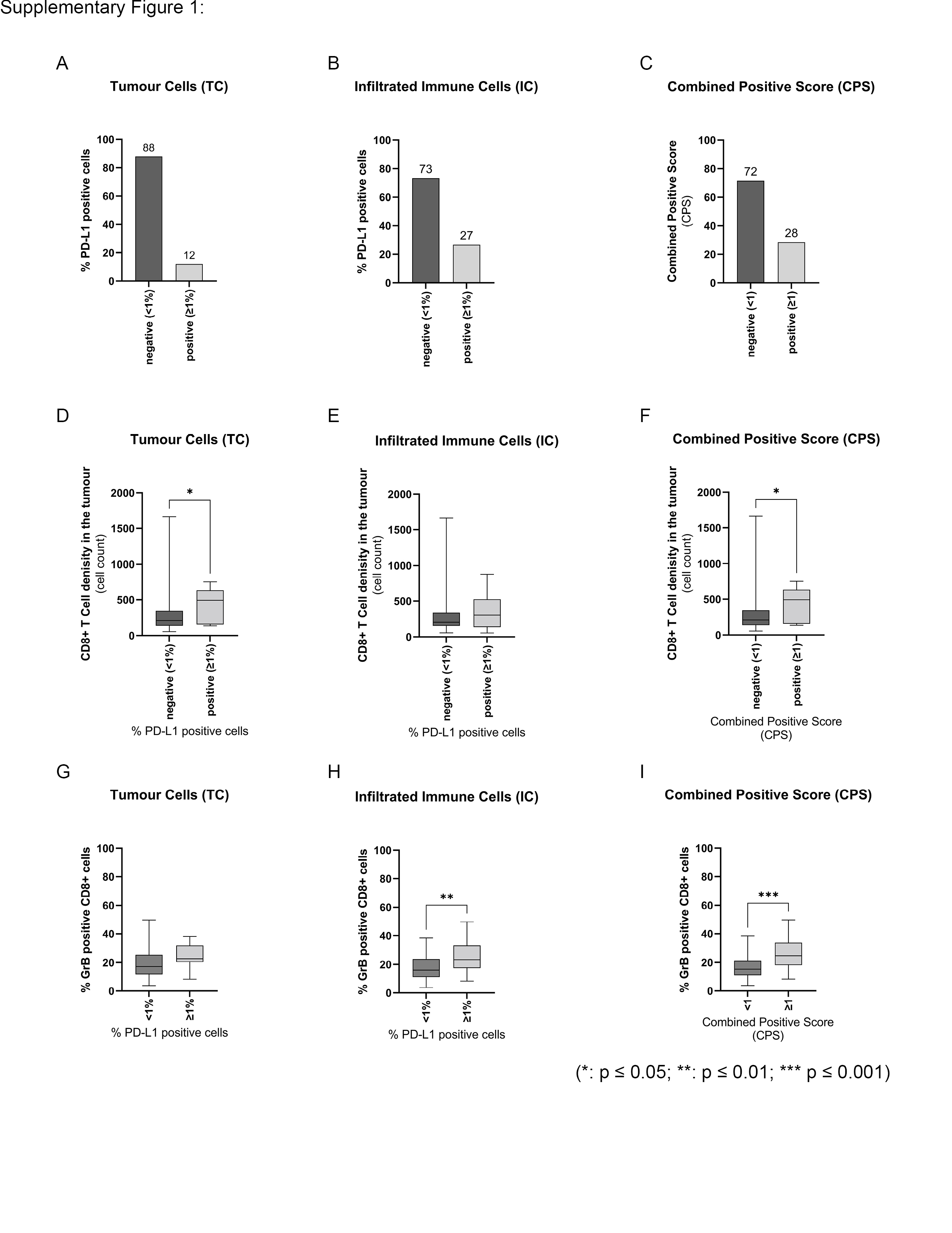

Supplement: Supplementary file 2 [file Image1.JPEG]

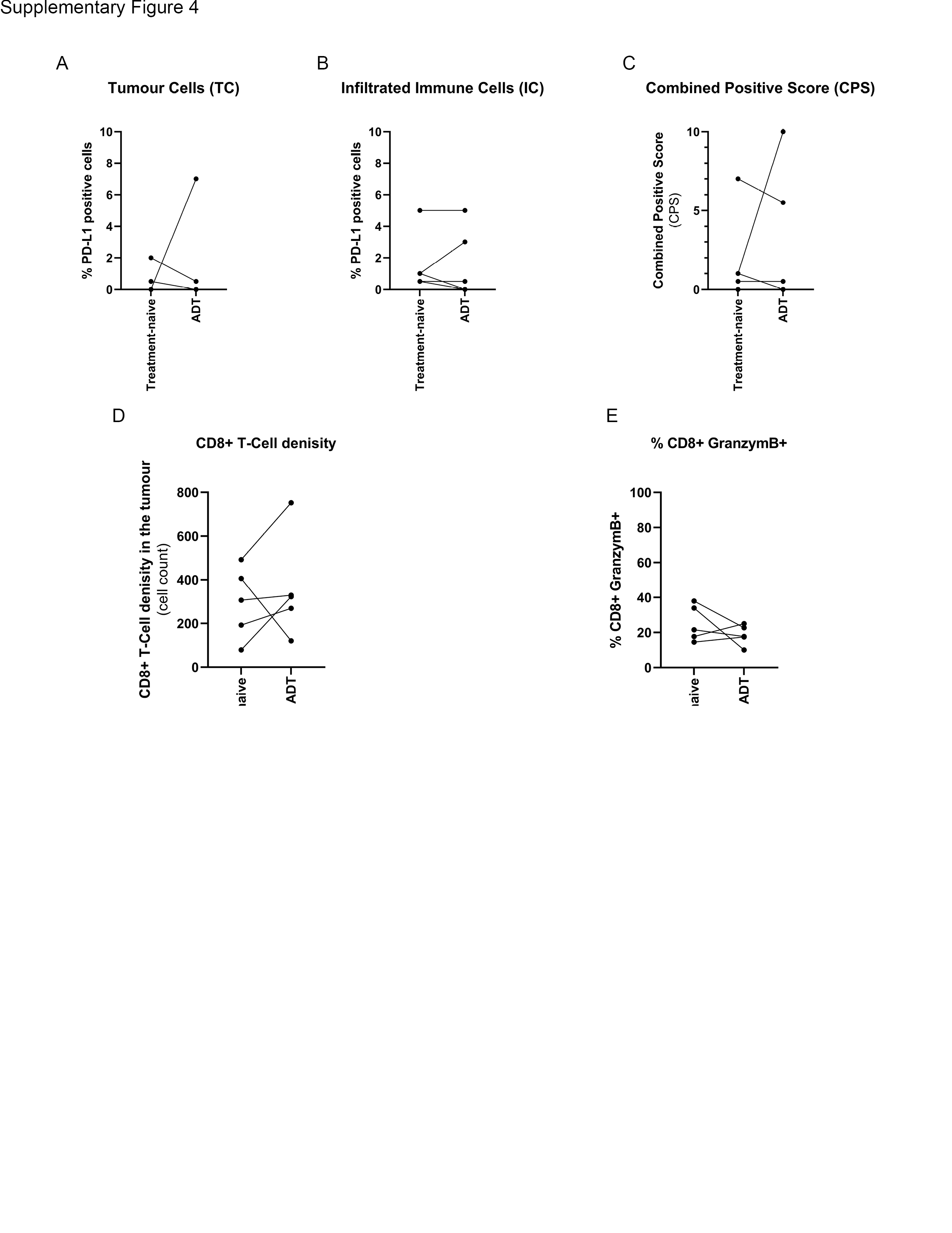

Supplement: Supplementary file 3 [file Image4.JPEG]

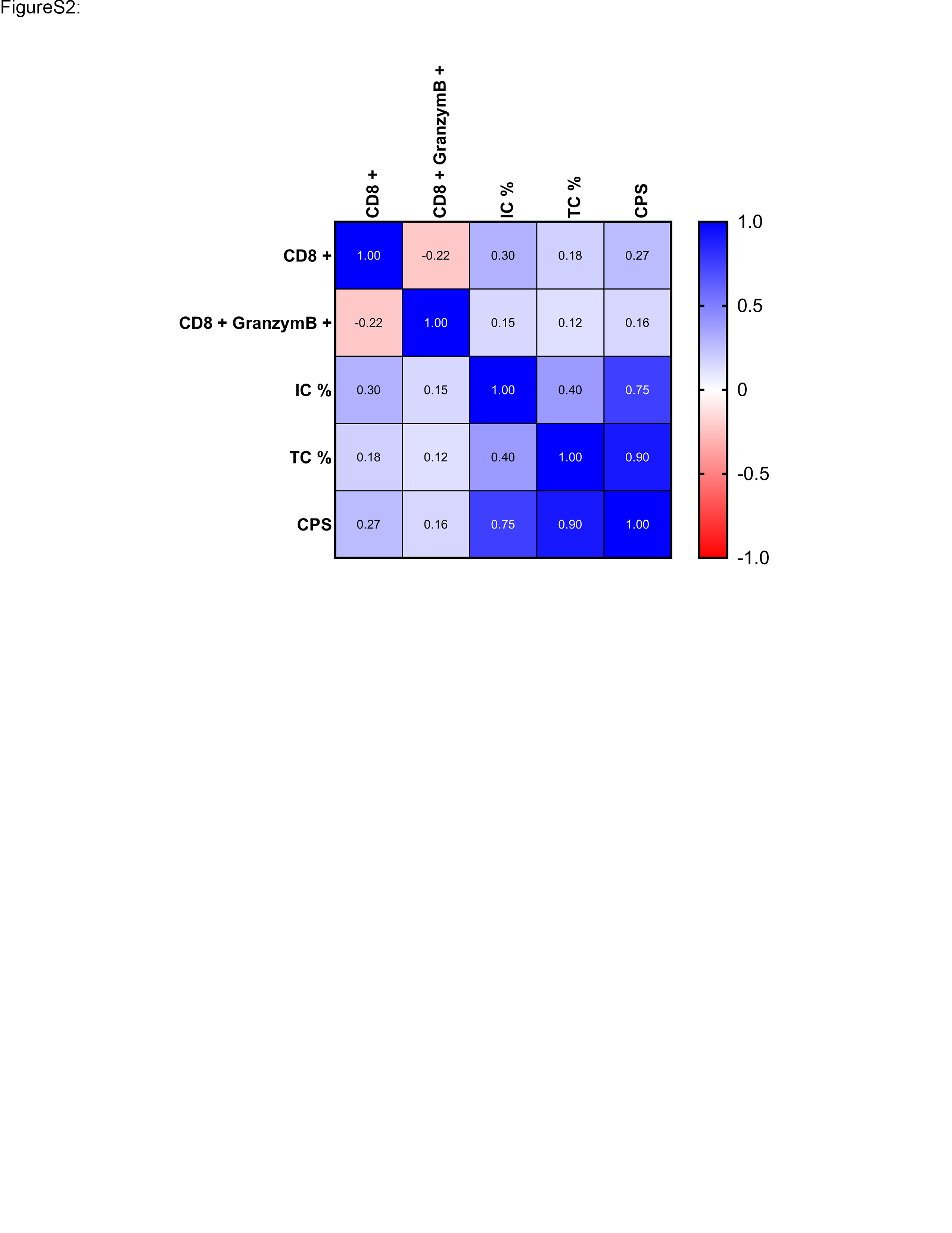

Supplement: Supplementary file 4 [file Image2.JPEG]
